# Improving Comfort in Obstetric Skills of Emergency Medicine Residents With Lecture- and Simulation-Based Training

**DOI:** 10.7759/cureus.77836

**Published:** 2025-01-22

**Authors:** Garrison Myers, Alicia Huckaby, Nancy Buderer

**Affiliations:** 1 Obstetrics and Gynecology, Mercy Health St. Vincent Medical Center, Toledo, USA; 2 Statistics, Nancy Buderer Consulting, LLC, Oak Harbor, USA

**Keywords:** collaborative training, emergency medicine, emergency medicine education, interdepartmental teaching, medical education, obstetrics, obstetric training, simulation

## Abstract

Objectives

The study aimed to determine whether obstetrician-led lecture- and simulation-based training improves Emergency Medicine (EM) residents’ comfort in managing complicated obstetric conditions.

Methods

Residents from Mercy St. Vincent Medical Center Emergency Medicine residency program in Toledo, Ohio participated in the study. Four clinical scenarios were chosen: shoulder dystocia, breech vaginal delivery, severe hypertensive disorder of pregnancy, and resuscitative hysterotomy. Participants attended a two-hour lecture series and subsequent simulation training with live-action situations for each chosen clinical scenario. Participants completed pre- and post-training surveys, which assessed comfort in performing the selected obstetric practices using the Likert Scale. Survey responses were analyzed for each item and presented by frequency count and percentage.

Results

Thirty-two EM residents completed a survey before the education and 25 of these residents completed a survey after the training. Before education, comfort levels performing obstetric procedures were low: three (9%) residents were comfortable knowing and performing maneuvers for shoulder dystocia, one (3%) for breech vaginal delivery, and one (3%) for resuscitative hysterotomy. Ten (31%) residents were comfortable managing severe hypertensive disorder. After education, the percentage of residents who reported being comfortable significantly increased (p<,0.05) in all clinical scenarios. Twenty-two (88%) residents strongly agreed that targeted lecture- and simulation-based training from an Obstetrician Gynecologist (OBGYN) will improve their comfort in assessing and treating complicated obstetric problems.

Conclusions

Obstetrician-led lecture- and simulation-based training can improve EM residents’ comfort in managing complicated obstetric conditions, and a collaborative, interdepartmental approach likely optimizes success in training EM residents.

## Introduction

Lecture- and simulation-based training are well-accepted educational modalities. Simulation-based training has been found to be a commonly used education tool when training high-risk, low-frequency medical events. It is studied across many facets of medicine, proving it to be useful [1]. A 2019 study of emergency medicine (EM) and obstetrics and gynecology (OBGYN) physicians demonstrated an increase in knowledge, skills, and a statistically significant increase in confidence following simulation-based training [2]. OBGYN residents have been found to have positive outcomes following specified training for high-risk obstetrics. In 2004, a group of OBGYN residents underwent detailed lecture- and simulation-based training for shoulder dystocia and performed significantly higher in overall performance and timeliness when compared to residents who did not receive training [3]. An additional 3-year observational study revealed a significant increase in OBGYN residents’ confidence when treating obstetric emergencies, specifically eclampsia, following simulation-based training [4]. The vast use of simulation in EM training has also been studied, more recently including interdisciplinary simulation sessions with OBGYN faculty and EM residents, with a preference for a curriculum that includes collaboration with the specialist [5].

EM physicians are trained to stabilize life-threatening conditions, including childbirth. EM physicians frequently encounter pregnant patients. In 2022, more than 3 million births took place in the United States, and approximately one in three pregnant people presented to the Emergency Department (ED) during pregnancy [6,7]. Although precise data on emergency department births is unknown in the United States, the rate of out-of-hospital births continues to rise. In 2012, 1.36% of births took place outside a hospital, increasing from 0.87% in 2004 [8]. Furthermore, the rate of complicated deliveries and obstetric emergencies continues to rise in the United States. The reported incidence of shoulder dystocia among vaginal deliveries has increased from approximately 0.2% to 3% [9]. Breech vaginal deliveries are a growing possibility as breech presentation occurs in approximately 3-4% of term pregnancies [10]. The frequency of hypertensive disorders of pregnancy also continues to rise while attributing to 16% of maternal deaths and is one of the leading causes of maternal and perinatal mortality worldwide. Preeclampsia specifically complicates 2-8% of pregnancies globally, with rates that increased by 25% between 1987 and 2004 [11,12]. Resuscitative hysterotomy (perimortem cesarean section) is one of the most challenging obstetric emergencies encountered by physicians and, although uncommon (2.78 per 100,000 pregnant persons), an attempt at the life-saving procedure has been found to have a survival rate as high as 58% [13].

As out-of-hospital births, obstetric emergencies, and complicated delivery rates increase, EM physicians are likely to experience complex obstetric conditions and situations. While a lecture-based approach is the traditional teaching model and simulation-based training is a more modern form of medical training, both are ideal approaches for solidifying the basic recognition, assessment, and management of complex obstetric situations. Although the EM residency curriculum includes obstetric training, this education initiative’s goal is to determine whether an obstetrician-led lecture- and simulation-based training improves EM residents’ comfort in managing complicated obstetric conditions.

## Materials and methods

A pre-post intervention study was performed at Mercy St. Vincent Medical Center (MSVMC) in Toledo, Ohio, USA, between October 2023 and January 2024. Participants included resident physicians of the MSVMC residency program. Forty-two residents were asked to participate and informed written consent was obtained. Inclusion criteria were active employment as an EM resident with the MSVMC EM residency program and exclusion criteria included previous formal obstetric training outside of current resident education. Participating EM residents were stratified by year of training and participated in a two-part course in complicated obstetrics. Four clinical scenarios were chosen: shoulder dystocia, breech vaginal delivery, severe hypertensive disorder of pregnancy, and resuscitative hysterotomy.

First, participants attended a two-hour lecture covering the four clinical scenarios. Lectures were written and co-presented by an OBGYN resident and attending physician and were based on the most current, evidence-based clinical recommendations from societal publications, including the American College of Obstetricians and Gynecologists (ACOG), The Society for Academic Specialists in General Obstetrics and Gynecology (SASGOG), and the Society of Maternal-Fetal Medicine (SMFM). The content of lectures was intentionally tailored to EM physicians. Objectives included the basic recognition, assessment, and management of the clinical scenarios and learning expectations were not set to the standard of an OBGYN specialist.

One week following the lecture, participants completed simulation training with live-action situations for each chosen clinical scenario. Simulation scenarios were written by an OBGYN resident and attending physicians and led by both EM attendings and an OBGYN resident. Learners encountered term pregnant patients presenting to the emergency department with complaints consistent with the clinical situation. Simulation training for shoulder dystocia, breech vaginal delivery, and resuscitative hysterotomy included maternal and fetal manikins, allowing hands-on participation in delivery maneuvers, surgical procedures, and resuscitative measures. Simulation of severe hypertensive disorder of pregnancy included a standardized patient actor with a display of simulated maternal vital signs and laboratory results. A debriefing session was performed after each simulation by the OBGYN resident and EM attending.

Participants completed pre- and post-training surveys. A Likert scale of 1 (strongly disagree) to 5 (strongly agree) was used (Table 1). The surveys included questions that assessed comfort in performing the selected obstetric practices before and after the training was completed. Surveys also included questions pertaining to a history of specified training by an OBGYN and general impressions of focused training by an OBGYN.

**Table 1:** Surveying Comfort in Obstetric Skills OBGYN: Obstetrician Gynecologist

1. Strongly Disagree	2. Disagree	3. Neutral	4. Agree	5. Strongly Agree
I believe I am unable to perform this task without significant training	I am uncomfortable attempting to perform this task and need more training	I am somewhat comfortable performing this task, but need additional training	I can comfortably perform this task	I can perform this task with ease, without assistance from an OBGYN

Survey responses were presented as frequency count and percentage, separately for each item before and after training. Using residents who completed both surveys, their change in comfort level was compared using McNemar’s two-sided tests. For the purpose of the analysis, “comfortable” is defined as a response of either “Agree - I can comfortably perform this task” or “Strongly Agree - I can perform this task with ease, without assistance from an OBGYN”. Data were analyzed with SAS v9.4 (SAS Institute, Cary, USA).

## Results

EM residents participated in the education. Thirty-two completed a survey before the education (13 Post-Graduate Year (PGY)-1, 10 PGY-2, 9 PGY-3) and 25 of these residents completed a survey after the training. No residents were excluded from the study. Residents had little to no prior patient encounters with shoulder dystocias (24 (75%) reported no cases), breech vaginal deliveries (25 (78%) none), or resuscitative hysterotomies (28 (88%) none) (Table 2). Before education, comfort levels performing obstetric procedures were low: three (9%) residents were comfortable knowing and performing maneuvers for shoulder dystocia; one (3%) was comfortable performing breech vaginal delivery; and one (3%) was comfortable performing resuscitative hysterotomy (Table 2). Ten (31%) residents were comfortable managing severe hypertensive disorder. Residents attributed their comfort level to a lack of training in the areas of shoulder dystocia (27 (84%) agreed or strongly agreed their training lacked) and breech delivery (27 (84%)) (Table 2). Lack of prevalence was also a factor that influenced comfort in the areas of shoulder dystocia (26 (81%) residents agreed or strongly agreed), breech delivery (23 (72%)), and severe hypertensive disorder (19 (59%)) (Table 2).

**Table 2:** Survey responses before and after education PGY: Post-Graduate Year; OBGYN: Obstetrician Gynecologist

	Before (n=32)	After (n=25)
Q1: Residency Year		
PGY 1	13 (40.6%)	10 (40.0%)
PGY 2	10 (31.3%)	8 (32.0%)
PGY 3	9 (28.1%)	7 (28%)
Other	0 (0%)	0 (0%)
Q2: Comfortable recognizing & diagnosing shoulder dystocia		
Strongly Disagree	3 (9.4%)	0 (0%)
Disagree	15 (46.9)	0 (0%)
Neutral	9 (28.1%)	1 (4.0%)
Agree	5 (15.6%)	18 (72.0%)
Strongly Agree	0	6 (24.0%)
Q3: Comfortable knowing & performing maneuvers for shoulder dystocia		
Strongly Disagree	9 (28.1%)	0 (0%)
Disagree	13 (40.6%)	0 (0%)
Neutral	7 (21.9%)	4 (16.0%)
Agree	3 (9.4%)	15 (60.0%)
Strongly Agree	0 (0%)	6 (24.0%)
Q4: How many shoulder dystocias encountered		
None	24 (75.0%)	20 (80.0%)
3 or less	8 (25%_	4 (16.0%)
3-5	0 (0%)	1 (4.0%)
More than 5	0 (0%)	0 (0%)
Q5: Comfort with shoulder dystocia due to lack of training		
Strongly Disagree	0 (0%)	N.A.
Disagree	2 (6.3%)	N.A.
Neutral	3 (9.4%)	N.A.
Agree	16 (50.0%)	N.A.
Strongly Agree	11 (34.4%)	N.A.
Q6: Comfort with shoulder dystocia due to lack of prevalence		
Strongly Disagree	0 (0%)	N.A.
Disagree	2 (6.3%)	N.A.
Neutral	4 (12.5%)	N.A.
Agree	16 (50.0%)	N.A.
Strongly Agree	10 (31.3%)	N.A.
Q7/Q5: Comfortable recognizing breech vaginal delivery		
Strongly Disagree	2 (6.3%)	0 (0%)
Disagree	6 (18.8%)	0 (0%)
Neutral	8 (25.0%)	1 (4.0%)
Agree	14 (43.8%)	13 (52.0%)
Strongly Agree	2 (6.3%)	11 (44.0%)
Q8/Q6: Understand risks of breech delivery		
Strongly Disagree	0 (0%)	0 (0%)
Disagree	3 (9.4%)	0 (0%)
Neutral	8 (25.0%)	1 (4.0%)
Agree	19 (59.4%)	15 (60.0%)
Strongly Agree	2 (6.3%)	9 (36.0%)
Q9/Q7: Comfortable performing breech vaginal delivery without an OBGYN		
Strongly Disagree	20 (62.5%)	0 (0%)
Disagree	12 (37.5%)	1 (4.0%)
Neutral	0 (0%)	17 (68.0%)
Agree	0 (0%)	6 (24.0%)
Strongly Agree	0 (0%)	1 (4.0%)
Q10/Q8: Comfortable performing maneuvers of breech vaginal delivery		
Strongly Disagree	16 (50.0%)	0 (0%)
Disagree	11 (34.4%)	0 (0%)
Neutral	4 (12.5%)	6 (24.0%)
Agree	1 (3.1%)	14 (56.0%)
Strongly Agree	0 (0%)	5 (20.0%)
Q11/Q9: Number breech vaginal deliveries encountered		
None	25 (78.1%)	21 (84.0%)
3 or less	7 (21.9%)	4 (16.0%)
3-5	0 (0%)	0 (0%)
More than 5	0 (0%)	0 (0%)
Q12: Comfort managing breech due to lack of training		
Strongly Disagree	0 (0%)	N.A.
Disagree	1 (3.1%)	N.A.
Neutral	4 (12.5%)	N.A.
Agree	15 (46.9%)	N.A.
Strongly Agree	12 (37.5%)	N.A.
Q13: Comfort managing breech due to lack of prevalence		
Strongly Disagree	0 (0.0%)	N.A.
Disagree	4 (12.5%)	N.A.
Neutral	5 (15.6%)	N.A.
Agree	15 (46.9%)	N.A.
Strongly Agree	8 (25.0%)	N.A.
Q14/Q10: Understand diagnostic criteria of severe hypertensive disorders of pregnancy		
Strongly Disagree	0 (0.0%)	0 (0.0%)
Disagree	3 (9.4%)	0 (0.0%)
Neutral	4 (12.5%)	1 (4.0%)
Agree	18 (56.3%)	9 (36.0%)
Strongly Agree	7 (21.9%)	15 (60.0%)
Q15/Q11: Comfortable performing initial work-up for severe hypertensive disorder of pregnancy		
Strongly Disagree	0 (0.0%)	0 (0.0%)
Disagree	1 (3.1%)	0 (0.0%)
Neutral	3 (9.4%)	0 (0.0%)
Agree	19 (59.4%)	5 (20.0%)
Strongly Agree	9 (28.1%)	20 (80.0%)
Q16/Q12: Comfortable stabilizing severe hypertensive disorder of pregnancy		
Strongly Disagree	0 (0.0%)	0 (0.0%)
Disagree	1 (3.1%)	0 (0.0%)
Neutral	9 (28.1%)	0 (0.0%)
Agree	16 (50.0%)	7 (28.0%)
Strongly Agree	6 (18.8%)	18 (72.0%)
Q17/Q13: Comfortable managing severe hypertensive disorder without an OBGYN		
Strongly Disagree	1 (3.1%)	0 (0%)
Disagree	7 (21.0%)	1 (4.0%)
Neutral	14 (43.8%)	1 (4.0%)
Agree	8 (25.0%)	15 (60.0%)
Strongly Agree	2 (6.3%)	8 (32.0%)
Q18/Q14: Number severe hypertensive disorders encountered		
None	5 (15.6%)	1 (4.0%)
1-5	11 (34.4%)	9 (36.0%)
5-10	11 (34.4%)	8 (32.0%)
>10	5 (15.6%)	7 (28.0%)
Q19: Comfort managing severe hypertensive disorder due to lack of training		
Strongly Disagree	1 (3.1%)	N.A.
Disagree	6 (18.8%)	N.A.
Neutral	6 (18.8%)	N.A.
Agree	17 (53.1%)	N.A.
Strongly Agree	2 (6.2%)	N.A.
-/Q15: Comfort managing severe hypertensive disorder has improved with specified training, despite the prevalence of disease in my field		
Strongly Disagree	N.A.	0 (0%)
Disagree	N.A.	0 (0%)
Neutral	N.A.	0 (0%)
Agree	N.A.	6 (24.0%)
Strongly Agree	N.A.	19 (76.0%)
Q20/Q16: Comfortable knowing when resuscitative hysterotomy is indicated		
Strongly Disagree	2 (6.3%)	0 (0%)
Disagree	5 (15.6%)	0 (0%)
Neutral	5 (15.6%)	2 (8.0%)
Agree	16 (50.0%)	7 (28.0%)
Strongly Agree	4 (12.5%)	16 (64.0%)
Q21/Q17: Understand materials for performing resuscitative hysterotomy		
Strongly Disagree	4 (12.5%)	0 (0%)
Disagree	5 (15.6%)	0 (0%)
Neutral	9 (28.1%)	0 (0%)
Agree	10 (31.3%)	8 (32.0%)
Strongly Agree	4 (12.5%)	17 (68.0%)
Q22/Q18: Comfortable performing resuscitative hysterotomy		
Strongly Disagree	13 (40.6%)	0 (0%)
Disagree	11 (34.4%)	1 (4.0%)
Neutral	7 (21.9%)	9 (36.0%)
Agree	1 (3.1%)	15 (60.0%)
Strongly Agree	0 (0%)	0 (0%)
Q23/Q19: Understand resources after performing resuscitative hysterotomy		
Strongly Disagree	6 (18.8%)	0 (0%)
Disagree	9 (28.1%)	0 (0%)
Neutral	10 (31.3%)	1 (4.0%)
Agree	4 (12.5%)	16 (64.0%)
Strongly Agree	3 (9.4%)	8 (32.0%)
Q24/20: Number or resuscitative hysterotomies encountered		
None	28(87.5%)	23 (92.0%)
3 or less	4 (12.5%)	2 (8.0%)
3-5	0 (0%)	0 (0%)
More than 5	0 (0%)	0 (0%)
Q25/Q21: Ever been lectured or participated in simulation by OBGYN resident or attending on these topics		
Yes	21 (65.6%)	25 (100%)
No	11 (34.4%)	0 (0%)
Q26/Q22: Targeted lecture-based training from an OBGYN will improve my comfort in assessing and treating complicated obstetric problems		
Strongly Disagree	0 (0%)	0 (0%)
Disagree	0 (0%)	0 (0%)
Neutral	1 (3.1%)	0 (0%)
Agree	12 (37.5%)	3 (12.0%)
Strongly Agree	19 (59.4%)	22 (88.0%)
Q27/Q23: Targeted simulation-based training from an OBGYN will improve my comfort in assessing and treating complicated obstetric problems		
Strongly Disagree	0 (0%)	0 (0%)
Disagree	0 (0%)	0 (0%)
Neutral	0 (0%)	1 (4.0%)
Agree	11 (34.4%)	2 (8.0%)
Strongly Agree	21 (65.6%)	22 (88.0%)

**Figure 1:**
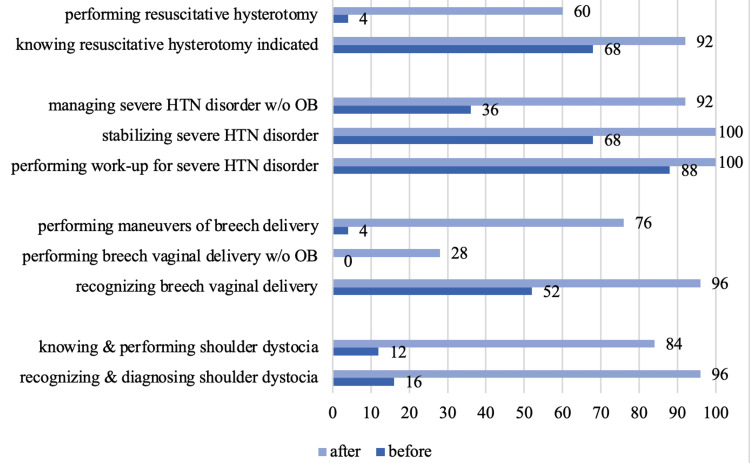
Increase in comfort level after training OB: Obstretician; HTN: hypertensive

Twenty-two (88%) residents strongly agreed that targeted lecture- and simulation-based training from an OBGYN will improve their comfort in assessing and treating complicated obstetric problems. After education, the percentage of residents who reported being comfortable significantly increased (p<0.05) in all clinical scenarios (Table 3, Figure 1). 

**Table 3:** Change in comfort before and after education Data are presented as the percentage of residents who agreed or strongly agreed with each statement among the 25 residents who completed both a pre and post survey.  P-value is from McNemar’s two-sided test. OBGYN: Obstetrician Gynecologist.

	Before (n=25)	After (n=25)	p-value
Q2: Comfortable recognizing & diagnosing shoulder dystocia	16%	96%	<0.001
Q3: Comfortable knowing & performing maneuvers for shoulder dystocia	12%	84%	<0.001
Q7 / Q5: Comfortable recognizing breech vaginal delivery	52%	96%	<0.001
Q8 / Q6: Understand risks of breech vaginal delivery	72%	96%	0.03
Q9 / Q7: Comfortable performing breech vaginal delivery without an OBGYN	0%	28%	n.a.
Q10 / Q8: Comfortable performing maneuvers of breech vaginal delivery	4%	76%	<0.001
Q14 / Q10: Understand diagnostic criteria of severe hypertensive disorders of pregnancy	80%	96%	0.1
Q15 / Q11: Comfortable performing initial work-up for severe hypertensive disorder of pregnancy	88%	100%	n.a.
Q16 / Q12: Comfortable stabilizing severe hypertensive disorder of pregnancy	68%	100%	n.a.
Q17 / Q13: Comfortable managing severe hypertensive disorder without an OBGYN	36%	92%	<0.001
Q20 / Q16: Comfortable knowing when resuscitative hysterotomy is indicated	68%	92%	0.03
Q21 / Q17: Understand materials for performing resuscitative hysterotomy	44%	100%	n.a.
Q22 / Q18: Comfortable performing resuscitative hysterotomy	4%	60%	<0.001
Q23 / Q19: Understand resources after performing resuscitative hysterotomy	24%	96%	<0.001

## Discussion

Previous literature supports the use of lecture- and simulation-based training for educating both OBGYN and EM residents in obstetric practices [2-5]. In this study, obstetrician-led training of EM residents resulted in statistically significant improved comfort in all clinical scenarios (Figure 1). Participants reported improved comfort in the recognition and management of shoulder dystocia, breech vaginal delivery, and resuscitative hysterotomy (p<0.001) and improved comfort in treating severe hypertensive disorders of pregnancy without assistance from an obstetrician (p<0.001).

Simulation-based education is often used for training in high-risk, low-frequency events [1]. For the EM Physician, obstetric emergencies and complicated deliveries fall in this category. Although one-third of pregnant people visit the ED, the prevalence of obstetric complications is low compared to other presenting illnesses, such as cardiopulmonary complaints or injuries [7]. In this study, at least 24 (75%) EM residents reported never encountering three of the four clinical scenarios. This data, along with the statistically significant improved comfort across all participants, supports the necessity of additional focused obstetric training in the EM curriculum.

Certain obstetric complications have higher incidence rates, such as hypertensive disorders of pregnancy [11,12]. This study suggests a relationship between EM residents’ comfort level and differing rates of obstetric complications. Prior to completing training, the pre-test survey demonstrated higher levels of comfort in assessing and treating severe hypertensive disorders of pregnancy compared to other obstetric conditions or situations. Prior to the training, 25 (78%) participants understood the diagnostic criteria, 28 (88%) were comfortable performing the initial workup, and 22 (68%) reported being comfortable stabilizing a patient with a severe hypertensive disorder. Comparing these pre-test responses with those of shoulder dystocia, only five (16%) participants reported being comfortable recognizing and three (9%) comfortable performing maneuvers to reduce a shoulder dystocia. It can be theorized that the increased level of pre-test comfort in assessing and treating severe hypertensive disorders of pregnancy is due to the increased prevalence of the disease. Prior to training, 27 (84%) residents reported having previously encountered severe hypertensive disorders, compared to shoulder dystocia, in which eight (25%) participants reported previously encountering and none had encountered more than three instances. Despite the reported increased pre-training comfort in treating severe hypertensive disorders of pregnancy, the pre-test also revealed only 10 (31%) residents felt comfortable managing this disease without assistance from an obstetrician. Following formal training, post-test surveys demonstrated that 23 (92%) residents were comfortable managing severe hypertensive disorders without assistance from an obstetrician (p < 0.001), and 25 (100%) agreed their comfort improved despite the prevalence of the disease. This suggests that although simulation-based training is often used for high-risk, low-frequency events, there is benefit in obstetrician-led lecture- and simulation-based training in complicated obstetrics regardless of prevalence. However, the significantly lower initial comfort levels of less common obstetric complications (shoulder dystocia, breech vaginal delivery, and resuscitative hysterotomy) can suggest more benefit in prioritizing the lower frequency obstetric complications when conducting specialized training.

Comfort is likely affected by a resident’s level of training. A 2016 study showed only half of EM residents reported adequate exposure to obstetric emergencies. Their comfort level in the management of such emergencies was found to be low, however, comfort increased with advancing PGY level [14]. Although stratification of results by PGY level did not produce significant results and post-training comfort was similar across all PGY levels in our study, pre-training comfort and PGY level were found to be directly proportional. Overall, this data suggests beginning obstetrician-lead training as early as PGY-1 and sustained training throughout residency could continue to increase a resident’s comfort in assessing and managing complicated obstetrics, however, this needs to be further studied.

This study suggests a collaborative, interdepartmental approach optimizes success in training EM residents. Approximately one-third (34%) of participating EM residents reported never receiving formal lectures or training from an obstetrician prior to the study. Following training, 25 (100%) participants agreed that lecture- and simulation-based training by an obstetrician will continue to improve their comfort in the management of complicated obstetrics. OBGYN generalists not only have a specialized skill set and understanding of maternal and fetal morbidity and mortality but also may understand education deficits in EM training due to their consistent work with EM residents when acting as consultants. However, this does not mean they understand or appreciate the challenges inherent in EM practice. In comparison, EM faculty better understand educational topics relevant to their practice, the limited resources of their department, and the high-stakes, low-frequency encounters faced in the ED [15]. Therefore, coordinating across departments is key to creating a meaningful learning experience. In this study, the writing and planning of lectures and simulations was a collective effort between OBGYN and EM departments, with an attempt to avoid addressing topics outside the scope of EM. This method was successful, however, it should continue to be studied for additional refinement for varied teaching environments. The ultimate goal is to make the ED safer for pregnant people and this study supports the notion that collaboration between OBGYN and EM departments and obstetrician-led training of EM residents can advance the EM curriculum.

Many EM residency programs have a singular rotation dedicated to obstetrics, commonly completed in the PGY-1 year. The Accreditation Council for Graduate Medical Education (ACGME) requires two weeks of obstetric experience or 10 vaginal deliveries for a resident [16]. Although the current standard may establish a foundation of knowledge, it does not require exposure to complicated obstetrics or provide sustained exposure. It is unclear if this standard provides the experience necessary to achieve comfort in assessing and treating obstetric emergencies and complications. While there is minimal data demonstrating high-fidelity methods of teaching obstetric emergencies to EM trainees, this study provides support for obstetrician-led lecture- and simulation-based training to improve EM residents’ comfort in the defined areas. Future investigations are required to determine the relationship between such training and competency in complicated obstetrics. EM education initiatives might also benefit from studying post-training retention rates to discover the necessary frequency of training to solidify comfort, knowledge, and skill.

Limitations of this study include a single-center study with a small sample size of EM residents who all attend the same residency program. This study explores EM residents’ comfort in assessing and treating complicated obstetrics but does not determine competency, knowledge, or skill, and therefore, future studies are needed to determine relationship. Overall, this study supports the incorporation of obstetrician-led lecture- and simulation-based training into the EM resident curriculum to optimize training in managing complicated obstetrics and ensure a more comprehensive educational experience.

## Conclusions

Obstetrician-led lecture- and simulation-based training can improve EM residents’ comfort in managing complicated obstetric conditions. Future investigations should be considered to determine the relationship between such training and competency in complicated obstetrics. A collaborative, interdepartmental approach likely optimizes success in training EM residents, and some prefer a curriculum that includes collaboration with OBGYN colleagues for obstetrics-specific training.
